# Higher versus lower doses of ACE inhibitors, angiotensin-2 receptor blockers and beta-blockers in heart failure with reduced ejection fraction: Systematic review and meta-analysis

**DOI:** 10.1371/journal.pone.0212907

**Published:** 2019-02-28

**Authors:** Ricky D. Turgeon, Michael R. Kolber, Peter Loewen, Ursula Ellis, James P. McCormack

**Affiliations:** 1 Division of Cardiology, Faculty of Medicine and Dentistry, University of Alberta, Edmonton, Alberta, Canada; 2 Department of Family Medicine, University of Alberta, Edmonton, Alberta, Canada; 3 Faculty of Pharmaceutical Sciences, Collaboration for Outcomes Research & Evaluation, University of British Columbia, Vancouver, British Columbia, Canada; 4 Woodward Library, University of British Columbia, Vancouver, British Columbia, Canada; 5 Faculty of Pharmaceutical Sciences, University of British Columbia, Vancouver, British Columbia, Canada; California Health Sciences University, UNITED STATES

## Abstract

**Background:**

Current heart failure (HF) guidelines recommend titrating angiotensin-converting enzyme inhibitors (ACEIs)/angiotensin receptor blockers (ARBs) and beta-blockers (BBs) to target doses used in pivotal placebo-controlled randomized controlled trials (RCTs). Despite a number of RCTs comparing different doses (i.e. higher versus lower doses) of ACEIs, ARBs and BBs, the effects of higher versus lower doses on efficacy and safety remains unclear. For this reason, we performed a systematic review and meta-analysis to evaluate the efficacy and safety of higher versus lower doses of ACEIs, ARBs and BBs in patients with HFrEF.

**Methods:**

We searched MEDLINE, Embase and the Cochrane Central Register of Controlled Trials (CENTRAL) via Ovid from inception to April 25^th^, 2018 and opentrials.net and clinicaltrials.gov for relevant trials that compared different doses of medications in heart failure. We analyzed trials by drug class (ACEIs, ARBs, and BBs) for efficacy outcomes (all-cause mortality, cardiovascular mortality, all-cause hospitalizations, HF hospitalizations, HF worsening). For safety outcomes, we pooled trials within and across drug classes.

**Results:**

Our meta-analysis consisted of 14 RCTs. Using GRADE criteria, the quality of evidence for ACEIs and ARBs was assessed as generally moderate for efficacy and high for adverse effects, whereas overall quality for BBs was very low to low. Over ~2–4 years higher versus lower doses of ACEIs, ARBs or BBs did not significantly reduce all-cause mortality [ACEIs relative risk (RR) 0.94 (95% confidence interval 0.87–1.02)], ARBs RR 0.96 (0.87–1.04), BBs RR 0.25 (0.06–1.01)] or all cause hospitalizations [ACEIs relative risk (RR) 0.94 (95% confidence interval 0.86–1.02)], ARBs RR 0.98 (0.93–1.04), BBs RR 0.93 (0.39–2.24)]. However, all point estimates favoured higher doses. Higher doses of ARBs significantly reduced hospitalization for HF [RR 0.89 (0.80–0.99)– 2.8% ARR], and higher doses of ACEIs and ARBs significantly reduced HF worsening [RR 0.85 (0.79–0.92)– 5.1% ARR and 0.91 (0.84–0.99)– 3.2% ARR, respectively] compared to lower doses. None of the differences between higher versus lower doses of BBs were significant; however, precision was low. Higher doses of these medications compared to lower doses increased the risk of discontinuation due to adverse events, hypotension, dizziness, and for ACEIs and ARBs, increased hyperkalemia and elevations in serum creatinine. Absolute increase in harms for adverse effects ranged from ~ 3 to 14%.

**Conclusions:**

Higher doses of ACEIs and ARBs reduce the risk of HF worsening compared to lower doses, and higher doses of ARBs also reduce the risk of HF hospitalization but the evidence is sparse and imprecise. Higher doses increase the chance of adverse effects compared to lower doses. Evidence for BBs is inconclusive. These results support initially always starting at low doses of ACEIs/ARBs and only titrating the dose up if the patient tolerates dose increases.

## Introduction

Heart failure (HF) with reduced ejection fraction (HFrEF) is a prevalent condition with an overall poor prognosis.[1] The combination of an angiotensin-converting enzyme inhibitor (ACEI) or angiotensin-2 receptor blocker (ARB) plus a beta-blocker (BB) is first-line therapy for HFrEF management,[1],[2] as these medications reduce morbidity and mortality compared to placebo.[3],[4],[5] These results have led guideline authors to universally recommend starting these agents in most patients with (HFrEF).[1],[2]

The approach recommended by guidelines when initiating these medications is to start at a low-to-moderate dose and titrate as tolerated to the target doses used in placebo-controlled randomized controlled trials (RCTs).[1],[2] However, many patients are unable to achieve and maintain target doses due to adverse effects, with most patients only achieving ~50% of the target dose.[6] Despite a number of RCTs comparing different doses (i.e. higher versus lower doses) of ACEIs, ARBs and BBs, the effects of higher versus lower doses on efficacy and safety remains unclear. For this reason, we performed a systematic review and meta-analysis to evaluate the efficacy and safety of higher versus lower doses of ACEIs, ARBs and BBs in patients with HFrEF.

## Methods

### Design

Systematic review with meta-analysis in accordance with the Preferred Reporting Items for Systematic reviews and Meta-Analyses (PRISMA) statement.[7]

### Search strategy

We searched MEDLINE, Embase and the Cochrane Central Register of Controlled Trials (CENTRAL) via Ovid from inception to April 25^th^, 2018 using keywords and subject headings for the following concepts: heart failure, ACEI, ARB, BB, dose, and randomized controlled trial (see S1 Appendix for MEDLINE search strategy). We also searched opentrials.net and clinicaltrials.gov for relevant RCTs, and hand-searched bibliographies of included studies.

### Eligibility criteria and outcomes

We included parallel RCTs published in English evaluating different doses of the same drug within the class of ACEIs, ARBs, or BBs in patients with HFrEF as defined by study investigators. Eligible trials needed to report results for at least one of the following outcomes: all-cause mortality, cardiovascular mortality, all-cause hospitalizations, HF hospitalizations, HF worsening, serious adverse events, discontinuation due to adverse events, hypotension, lightheadedness/dizziness, hyperkalemia, renal dysfunction, and cough. While there is evidence some of these drug classes have dose-related hemodynamic and neurohormonal effects, we chose to only include trials whose clinical outcomes would likely be relevant to clinicians and patients.

### Study selection and data collection

Three authors (MK, PL, JM) screened candidate articles for inclusion, identified missing data, and consulted original publications. The same authors individually extracted crude outcome data from full-text reports into a standardized database. For trials comparing >2 doses, we included the results from only the lowest and highest dose arms, hereafter referred to as “higher dose” and “lower dose”. Discrepancies and missing data were resolved by group discussion, reference to the original publication, and additional independent adjudication by all authors.

### Risk of bias and quality of evidence assessment

Two authors (JM, PL) assessed individual trials for risk of bias using the Cochrane Collaboration Risk of Bias Tool.[8] For each trial, we classified trials as being at low, high, or unclear risk of bias in each of 5 domains: sequence generation, allocation concealment, blinding of participants and personnel, blinding of outcome assessors and incomplete outcome data. Disagreements were resolved by consensus with a third author (RT). We also rated the quality of evidence for individual outcomes from high to very low based on Grading of Recommendations Assessment, Development and Evaluation (GRADE) methodology, which incorporates risk of bias, consistency, directness, precision, and other considerations.[9]

### Synthesis of results and assessment of heterogeneity

We performed all analyses using Review Manager 5.3 (Cochrane Collaboration). We pooled trials by drug class studied at the study level and calculated risk ratios (RRs) with 95% confidence intervals (CIs), and absolute risk difference per 1000 patients treated over the follow-up period. For efficacy outcomes, we analyzed trials by drug class (ACEIs, ARBs, and BBs). For safety outcomes, we pooled trials within and across drug classes. All comparisons used high dose as the experimental intervention and low dose as the control intervention. We evaluated for statistical heterogeneity by visually inspecting forest plots and by calculating the I^2^ statistic. For efficacy outcomes, we reported fixed-effects model results for analyses with I^2^<50% and the random-effects model results for an I^2^≥50%. We reported all safety outcomes using fixed-effects models to minimize type 2 error.

## Results

### Included studies

**Fig 1:**
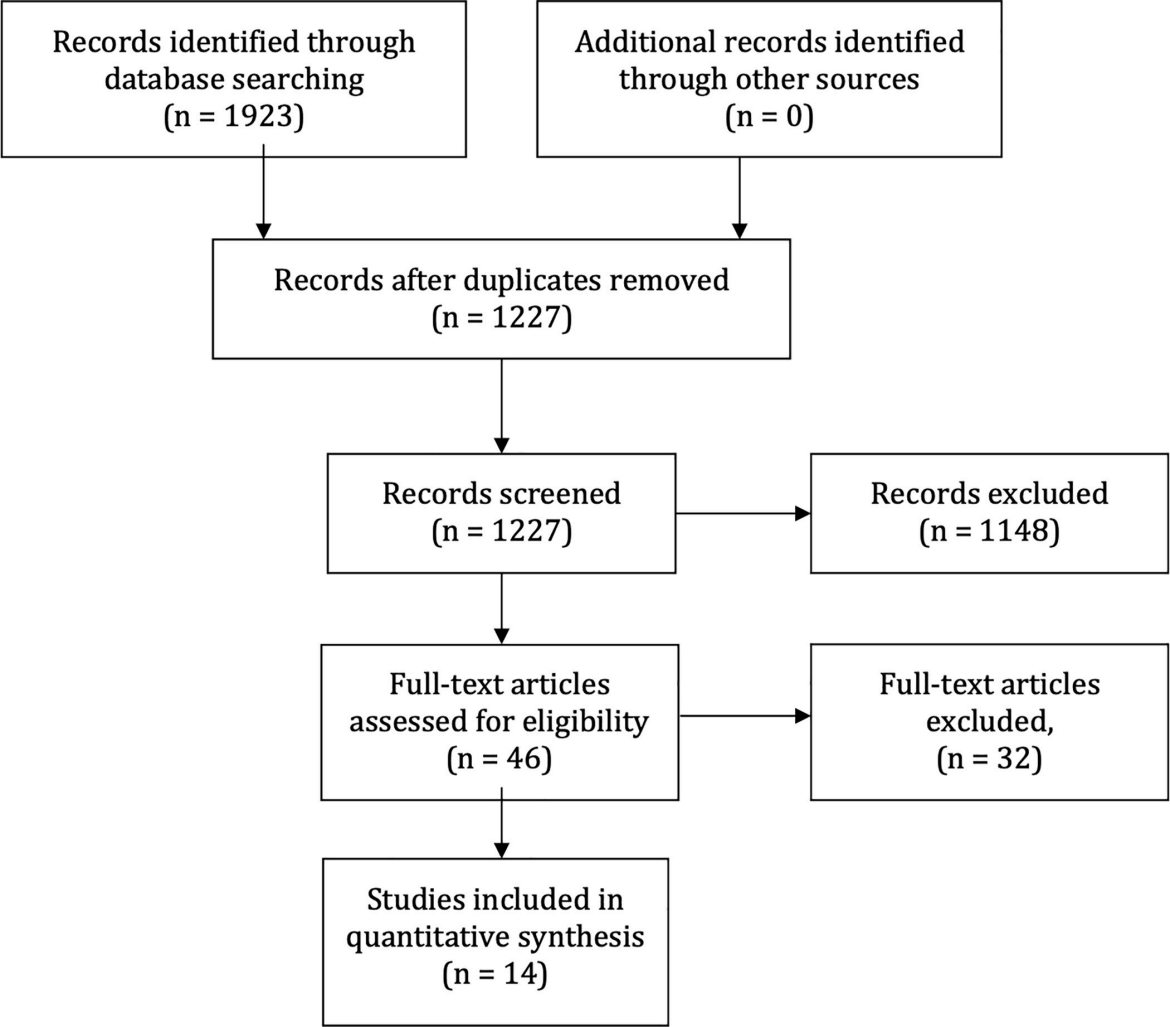
PRISMA flow diagram.

From 1923 articles identified, we included 14 RCTs with sample size ranging from 83 to 3846 participants (Fig 1). We identified evidence for the following interventions: ACEIs (7 studies, n = 5,817),[10],[11],[12],[13],[14],[15],[16] ARBs (3 studies, n = 4,908),[17],[18],[19] and BBs (4 studies, n = 1,018).[20],[21],[22],[23]

### Characteristics of included trials

Table 1 summarizes key characteristics of the included trials. At baseline, the mean age across trials ranged from 54–70 years, approximately 75% of participants were male, and the majority had NYHA functional class 2 or 3 symptoms. Twelve trials required reduced ejection fraction (EF) at baseline, ranging from <30% to <50%. Eight trials compared >2 doses; seven studies (50%) compared a 4-fold increase in dose (between the groups receiving the lowest and highest dose), 4 studies (29%) compared 7 to 16-fold differences in doses, and three studies (21%) compared a 2 to 3-fold difference in dose. The weighted mean follow-up duration across included studies was 34 months (ACEIs 28 months, ARBs 45 months, BBs 17 months). Three studies that accounted for 63% of patients in this meta-analysis had follow-up greater than 12 months.[10],[18],[21]

**Table 1:** Characteristics of included trials.

			Baseline											Study intervention			
Trial (year)	n	Follow-up duration, months	Mean age, y	Male, %	NYHA class, %			LVEF, %		ACEI or ARB	BB	MRA	Digoxin	Drug	Higher dose, mg/day	Lower dose, mg/day	Ratio
					II	III	IV	For inclusion	Mean								
**ACEIs**																	
ATLAS (1999)	3164	Mean 46 (range 39–58)	64	80	16	77	7	≤30	23	89	NR	NR	NR	Lisinopril	32.5 to 35	2.5 to 5	7–13
NETWORK (1998)	1532	6	70	64	65	33	2	None	20a	NR	11	NR	24	Enalapril	20	5	4
CHIPS (2000)	298	12	65	69	38	50	0	<45	NR	NR	26	NR	31	Captopril	100	50	2
HEDS (2000)	248	12	56	85	42	44	11	≤35	20	NR	NR	NR	92	Enalapril	60	20	3
CASSIS (1995)	248	3	58	83	25	56	19	<40	28	5	NR	NR	91	Spirapril	6	1.5	4
van Veldhuisen (1998)	244	3	61	77	77	23	0	<45	34	NR	NR	NR	38	Imidapril	10	2.5	4
Pacher (1996)	83	11	56	83	2	80	18	None	NR	100	NR	2	100	Enalapril	40	10	4
**ARBs**																	
HEAAL (2009)	3846	Mean 56 (IQR 41–66)	66	71	69	30	1	<40	33	NR	72	38	42	Losartan	150	50	3
STRETCH (1999)	844	3	62	68	81	19	0	30–40	39	59	NR	NR	42	Candesartan	16	4	4
Havranek (1999)	218	3	60	82	63	21	16	<40	24	NR	NR	NR	NR	Irbesartan	150	12.5	12
**BBs**																	
J-CHF (2013)	360	Mean 36	60	74	83	17	0	≤40	30	87	NR	NR	33	Carvedilol	20	2.5	8
MOCHA (1996)	345	6	60	76	46	52	2	≤35	23	94	NR	NR	92	Carvedilol	50	12.5	4
MUCHA (2004)	174	6–12	60	78	79	21	0	<40	30	76	NR	NR	67	Carvedilol	20	5	4
Bristow (1994)	139	3	54	61	42	55	0	<40	24	87	NR	NR	79	Bucindolol	200	12.5	16

ACEI: Angiotensin-converting enzyme inhibitor, ARB: Angiotensin-2 receptor blocker, BB: beta-blocker, IQR: Interquartile range, LVEF: Left ventricular ejection fraction, MRA: Mineralocorticoid receptor antagonist, NR: Not reported.

a Estimated from mean baseline left ventricular end-diastolic (LVEDD) and end-systolic dimensions (LVESD) using the equation LVEF = (LVEDD-LVESD) / LVEDD

### Risk of bias and quality assessment

**Fig 2:**
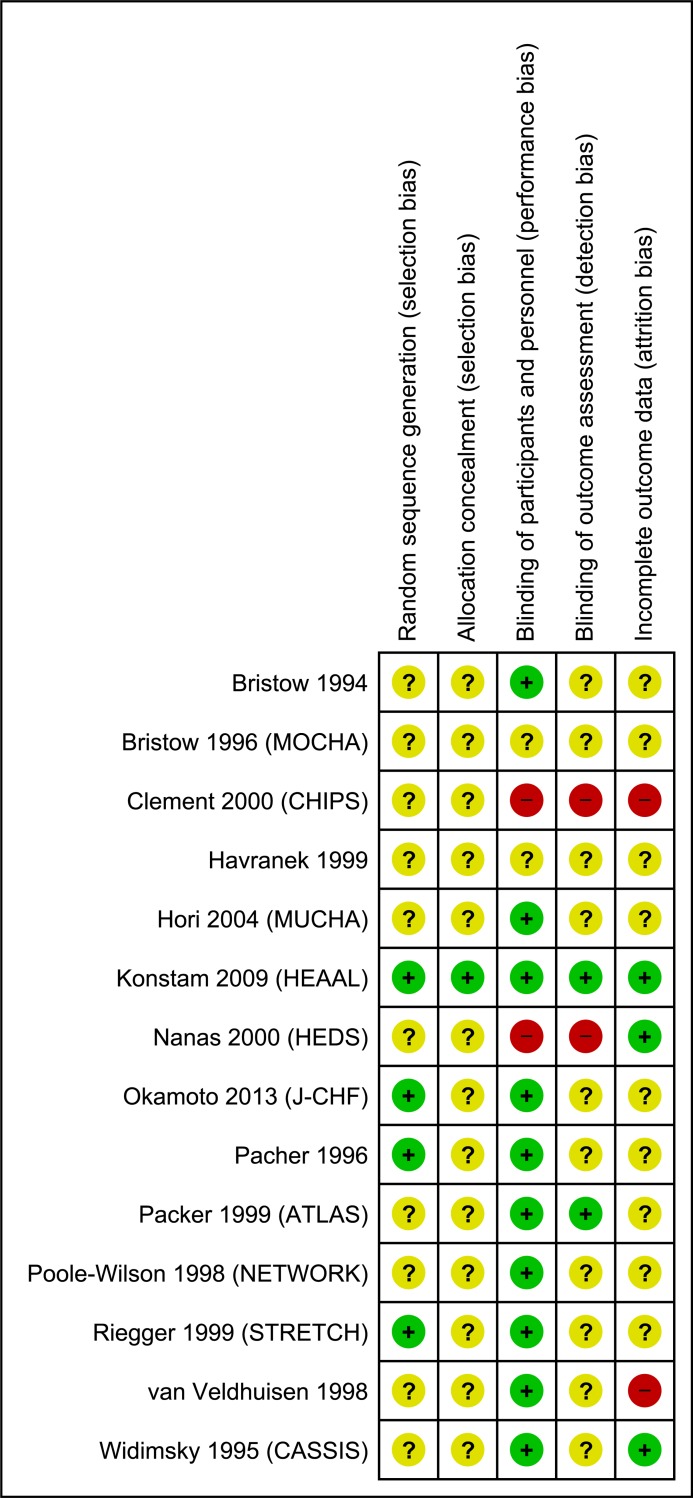
Risk of bias summary.

All trials except the HEAAL study had high or unclear risk of bias in at least 3 of the 5 domains (Fig 2). Quality of evidence for ACEIs and ARBs was generally moderate for efficacy and high for adverse effects, whereas overall quality for BBs was very low to low. Table 2 lists the quality of evidence for each outcome and reasons for downgrading. We did not assess for publication bias as the number of studies for each meta-analysis was insufficient according to accepted criteria.[24]

**Table 2:** GRADE summary of findings. Statistically significant results are shown in **bold text**.

	ACEIs			ARBs			BBs		
Outcome	Quality of evidence	Effect (follow-up range 3–58 months–mean 28 months)		Certain assessment	Effect (follow-up range 3–66 months–mean 45 months)		Certain assessment	Effect (follow-up range 3–36 months–mean 17 months)	
		RR (95% CI)	Absolute change, per 1000		RR (95% CI)	Absolute change, per 1000		RR (95% CI)	Absolute change, per 1000
Mortality									
All-cause	MODERATE ^1^	0.94 (0.87 to 1.02)	19 fewer (from 6 more to 40 fewer)	MODERATE ^1^	0.96 (0.87 to 1.04)	12 fewer (from 12 more to 40 fewer)	LOW ^2^	0.25 (0.06 to 1.01)	36 fewer (from 0 more to 45 fewer)
Cardiovascular	MODERATE ^1^	0.92 (0.85 to 1.01)	30 fewer (from 4 more to 55 fewer)	MODERATE ^1^	0.93 (0.83 to 1.04)	17 fewer (from 10 more to 42 fewer)	-	NR	NR
Hospitalization									
All-cause	MODERATE ^1^	0.94 (0.86 to 1.02)	22 fewer (from 7 more to 52 fewer)	MODERATE ^1^	0.98 (0.93 to 1.04)	11 fewer (from 23 more to 40 fewer)	LOW ^2^	0.93 (0.39 to 2.24)	8 fewer (from 66 fewer to 134 more)
Heart failure	LOW ^1^ ^3^	0.86 (0.56 to 1.32)	18 fewer (from 42 more to 58 fewer)	MODERATE ^1^	**0.89** **(0.80 to 0.99)**	**28 fewer** (from 3 to 52 fewer)	LOW ^2^	2.48 (0.60 to 10.28)	23 more (from 6 fewer to 143 more)
Heart failure worsening	HIGH	**0.85** **(0.79 to 0.92)**	**51 fewer** (from 27 to 71 fewer)	MODERATE ^1^	**0.91** **(0.84 to 0.99)**	**32 fewer** (from 4 to 57 fewer)	LOW ^2^	0.45 (0.11 to 1.86)	16 fewer (from 26 fewer to 26 more)
Adverse effects									
Leading to discontinuation	MODERATE ^1^	1.19 (0.97 to 1.46)	13 more (from 2 fewer to 31 more)	MODERATE ^1^	1.17 (0.94 to 1.45)	11 more (from 4 fewer to 30 more)	VERY LOW ^2^ ^4^	1.98 (0.37 to 10.62)	16 more (from 11 fewer to 162 more)
Hypotension	HIGH	**1.60** **(1.28 to 2.00)**	**30 more** (from 14 to 51 more)	HIGH	**1.40** **(1.15 to 1.72)**	**27 more** (from 10 to 49 more)	VERY LOW ^2^ ^4^	1.12 (0.35 to 3.53)	7 more (from 39 fewer to 152 more)
Dizziness	MODERATE ^3^	**1.39** **(1.22 to 1.59)**	**51 more** (from 29 to 77 more)	-	NR	NR	LOW ^1^ ^4^	**1.59** **(1.00 to 2.52)**	**142 more** (from 0 to 366 more)
Hyperkalemia	HIGH	**1.87** **(1.39 to 2.51)**	**24 more** (from 11 to 42 more)	MODERATE ^4^	**1.48** **(1.20 to 1.83)**	**33 more** (from 14 to 57 more)	**-**	NR	NR
Serum creatinine increase	HIGH	**1.46** **(1.18 to 1.81)**	**27 more** (from 10 to 47 more)	HIGH	**1.41** **(1.24 to 1.60)**	**62 more** (from 37 to 91 more)	**-**	NR	NR
Cough	MODERATE ^1^	**0.83** **(0.71 to 0.97)**	**23 fewer** (from 4 to 39 fewer)	-	NR	NR	-	NR	NR

ACEI: Angiotensin-converting enzyme inhibitor, ARB: Angiotensin-2 receptor blocker, BB: beta-blocker, NR: Not reported.

^1^ Rated down 1 category for serious imprecision

^2^ Rated down 2 categories for very serious imprecision

^3^ Rated down 1 category for serious inconsistency

^4^ Rate down 1 category for suspected selective outcome reporting bias

### Efficacy

**Fig 3:**
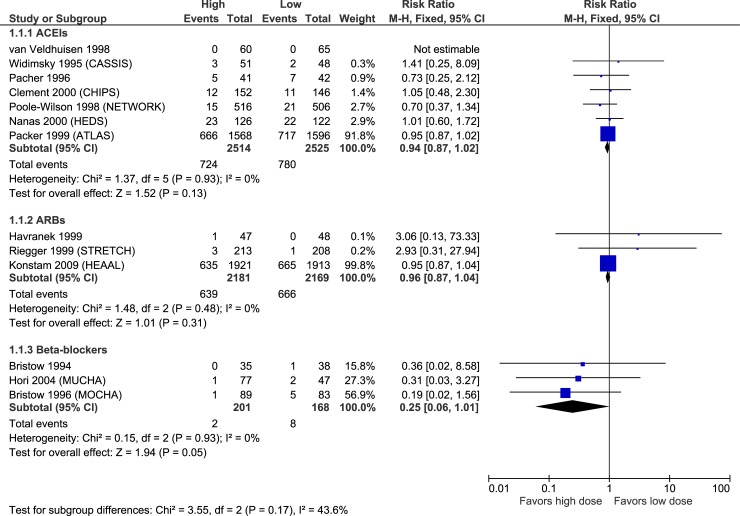
Forest plot of risk ratios (with 95% confidence intervals) of overall mortality for higher doses versus lower doses of ACE inhibitors, angiotensin-2 receptor blockers and beta-blockers in patients with heart failure with reduced ejection fraction.

**Fig 4:**
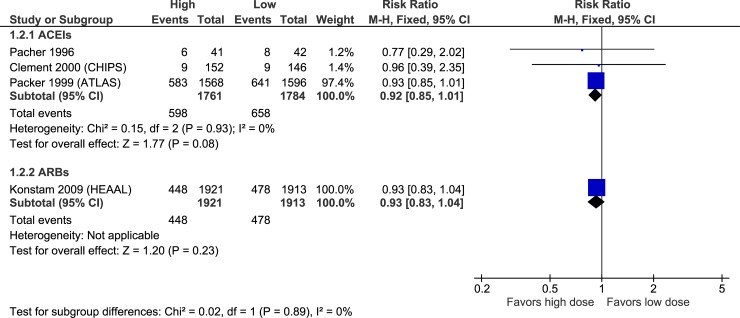
Forest plot of risk ratios (with 95% confidence intervals) of cardiovascular mortality for higher doses versus lower doses of ACE inhibitors, angiotensin-2 receptor blockers and beta-blockers in patients with heart failure with reduced ejection fraction.

**Fig 5:**
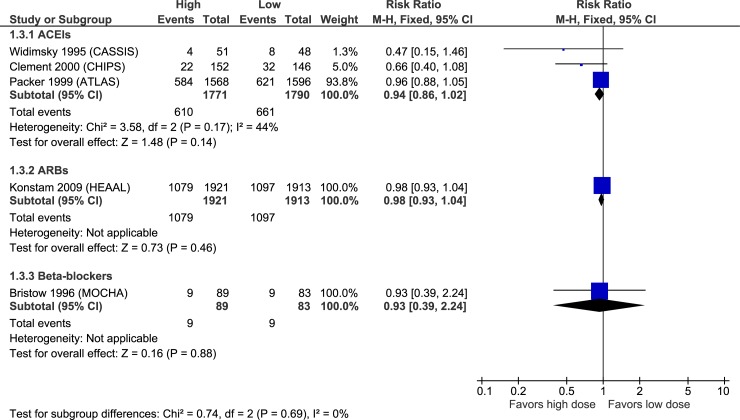
Forest plot of risk ratios (with 95% confidence intervals) of all-cause hospitalization overall mortality for higher doses versus lower doses of ACE inhibitors, angiotensin-2 receptor blockers and beta-blockers in patients with heart failure with reduced ejection fraction.

**Fig 6:**
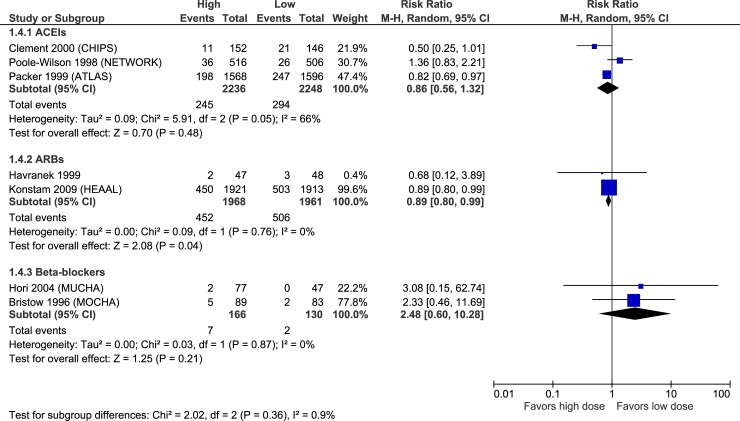
Forest plot of risk ratios (with 95% confidence intervals) of hospitalization for heart failure for higher doses versus lower doses of ACE inhibitors, angiotensin-2 receptor blockers and beta-blockers in patients with heart failure with reduced ejection fraction.

**Fig 7:**
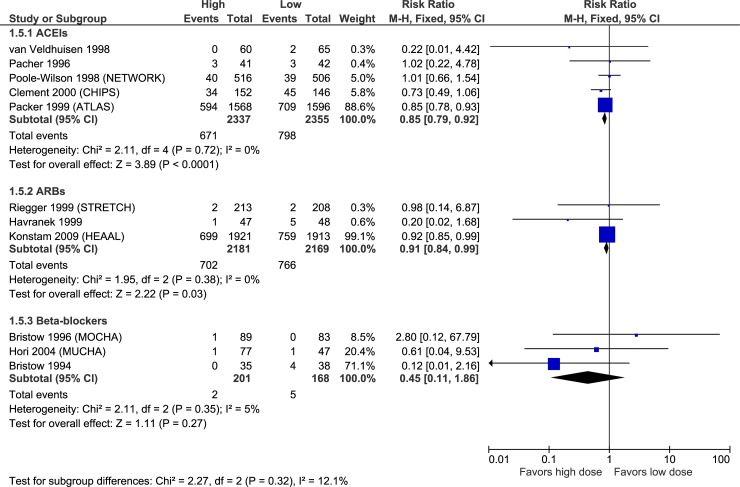
Forest plot of risk ratios (with 95% confidence intervals) of heart failure worsening for higher doses versus lower doses of ACE inhibitors, angiotensin-2 receptor blockers and beta-blockers in patients with heart failure with reduced ejection fraction.

**Fig 8:**
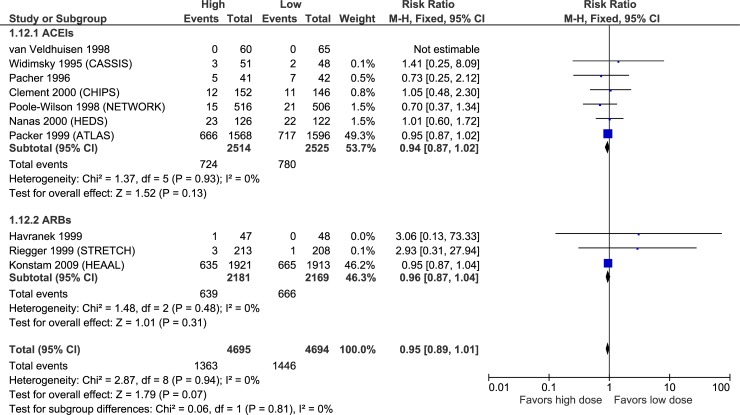
Forest plot of risk ratios (with 95% confidence intervals) of overall mortality (post-hoc analyses pooling studies of ACE inhibitors and angiotensin-2 receptor blockers) for higher doses versus lower doses in patients with heart failure with reduced ejection fraction.

**Fig 9:**
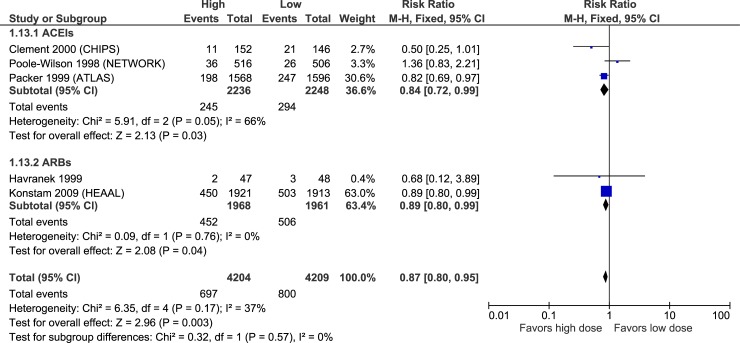
Forest plot of risk ratios (with 95% confidence intervals) of hospitalization for heart failure (post-hoc analyses pooling studies of ACE inhibitors and angiotensin-2 receptor blockers) for higher doses versus lower doses in patients with heart failure with reduced ejection fraction.

Figs 3–7 illustrate the meta-analyses for efficacy outcomes, and Table 2 summarizes the absolute and relative estimates for efficacy. Post-hoc analyses pooling studies of ACEIs and ARBs are shown in Figs 8 and 9.

### Adverse effects

**Fig 10:**
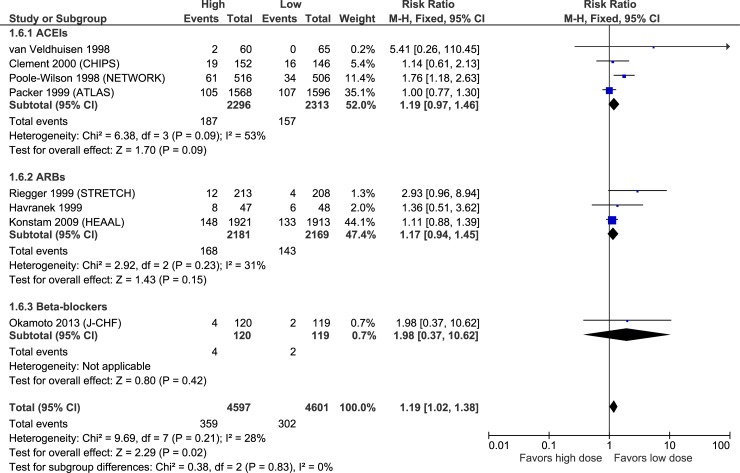
Forest plot of risk ratios (with 95% confidence intervals) of discontinuation for adverse effects for higher doses versus lower doses of ACE inhibitors, angiotensin-2 receptor blockers and beta-blockers in patients with heart failure with reduced ejection fraction.

**Fig 11:**
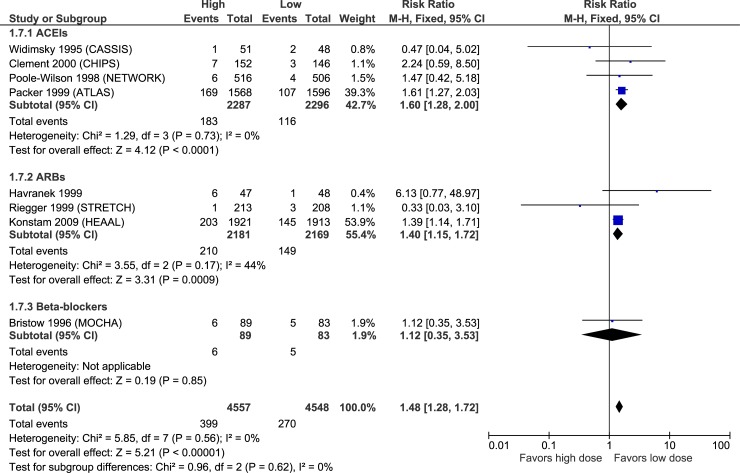
Forest plot of risk ratios (with 95% confidence intervals) of hypotension for higher doses versus lower doses of ACE inhibitors, angiotensin-2 receptor blockers and beta-blockers in patients with heart failure with reduced ejection fraction.

**Fig 12:**
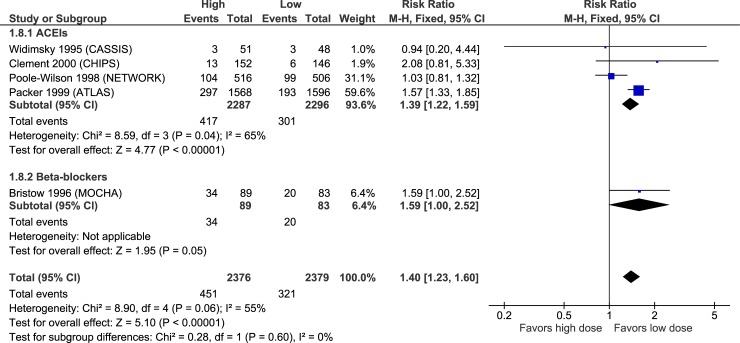
Forest plot of risk ratios (with 95% confidence intervals) of dizziness for higher doses versus lower doses of ACE inhibitors, angiotensin-2 receptor blockers and beta-blockers in patients with heart failure with reduced ejection fraction.

**Fig 13:**
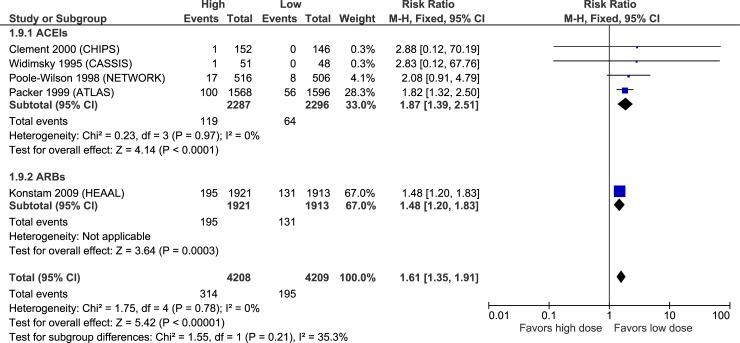
Forest plot of risk ratios (with 95% confidence intervals) of hyperkalemia for higher doses versus lower doses of ACE inhibitors, angiotensin-2 receptor blockers and beta-blockers in patients with heart failure with reduced ejection fraction.

**Fig 14:**
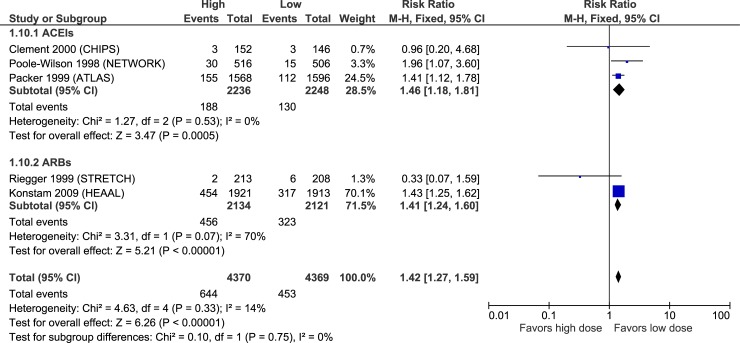
Forest plot of risk ratios (with 95% confidence intervals) of creatinine increases for higher doses versus lower doses of ACE inhibitors, angiotensin-2 receptor blockers and beta-blockers in patients with heart failure with reduced ejection fraction.

**Fig 15:**
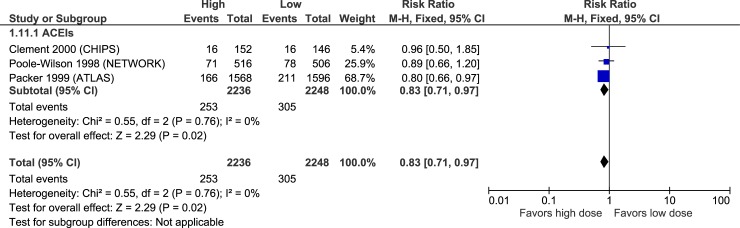
Forest plot of risk ratios (with 95% confidence intervals) of cough for higher doses versus lower doses of ACE inhibitors, angiotensin-2 receptor blockers and beta-blockers in patients with heart failure with reduced ejection fraction.

Figs 10–15 illustrate the meta-analyses for adverse effects, and Table 2 summarizes the absolute and relative estimates for adverse effects.

## Discussion

In this systematic review and meta-analysis of RCTs, higher doses of ACEIs were not statistically superior to the lower doses studied in terms of mortality or hospitalizations, but point estimates were in favour of higher doses. Higher doses of ACEIs statistically-significantly reduced the proportion of patients with episodes of worsening HF compared to lower doses. Similarly, higher doses of ARBs did not statistically reduce mortality or all-cause hospitalizations compared to lower doses, but point estimates were again in favour of higher doses. However, higher doses of ARBs did statistically reduced HF hospitalization and episodes of worsening HF compared to the lower doses studied. We did not find statistically-significant differences between higher and lower doses of BBs for any efficacy outcome, though results were imprecise due to few events, limiting the certainty of these findings. Higher doses increased the risk of discontinuation due to adverse events, hypotension, dizziness, and for ACEIs and ARBs, increased hyperkalemia and elevations in serum creatinine compared to lower doses.

Based on these pooled results, treating 1000 patients with higher versus lower doses of ACEIs over a mean of 28 months would result in 51 fewer patients experiencing worsening HF and 23 fewer patients experiencing cough (possibly related to the lower incidence of worsening HF). These benefits would be offset by 30–51 more patients experiencing hypotension or dizziness, and 24–27 patients having laboratory abnormalities including hyperkalemia or an increase in serum creatinine. Treating 1000 patients with higher versus lower doses of ARBs over a mean of 45 months would lead to 28–32 fewer cases of HF hospitalization or worsening, 27 more patients experiencing symptomatic hypotension, 33 more patients with hyperkalemia and 62 more patients with increased serum creatinine. For BBs the data is too imprecise to estimate efficacy, but suggest 142 more cases of dizziness per 1000 patients treated with higher doses versus lower doses over 17 months.

Our results differ somewhat from those of a recent meta-analysis comparing higher versus lower doses of ACEIs/ARBs by Khan et al.[25] These authors reported that when the data from trials evaluating ACEIs and ARBs are combined, higher doses showed a reduced risk of all-cause mortality, but no difference in hospitalizations for HF or discontinuations due to adverse effects when compared to lower doses [25] Our meta-analysis included 4 additional trials not included in the Khan meta-analysis. In our post-hoc analysis pooling ACEIs plus ARBs for the outcome of all-cause mortality, we did not find a statistically significant difference between higher and lower doses. However, our results are quantitatively nearly identical (confidence interval 0.89–1.01 versus 0.89–1.00), and the apparent difference in the report of findings can be solely explained by dogmatic adherence to arbitrary dichotomous statistical significant thresholds.[26] Finally, for safety outcomes we used fixed-effects meta-analyses to minimize type 2 error which might account for some of the differences reported.

### Applying the evidence

Given that the benefit of ACEIs, ARBs and BBs accrue over months, yet adverse effects typically occur early after starting or increasing doses, clinicians should initiate these agents at a low dose and titrate them gradually while assessing tolerability. American and Canadian HFrEF guidelines recommend this approach, with a goal of titrating the dose every 2–4 weeks as an outpatient to target doses used in the landmark placebo-controlled trials.[1],[2] Missing from these guidelines, however, is a discussion of the benefits and harms of dose titration. Ideally, HF guidelines would stress the important role that shared decision-making plays in this process, particularly when a patient experiences adverse effects and must decide on the value of maintaining their current dose or continuing to up-titrate. Clinicians can use the results of this review, as summarized in Table 2, to discuss what is known about the benefits and harms of higher versus lower doses of these agents, as well areas of uncertainty about clinically-important outcomes.

### Limitations

Our meta-analysis has limitations stemming primarily from the included trials. First, included trials did not always report all outcomes of interest, especially for BBs, limiting available data and decreasing the certainty in pooled estimates. Furthermore, trials seldom reported the definitions for adverse effects such as hypotension and increase in serum creatinine, and it is therefore difficult to put their clinical significance into context without knowledge of their severity or consequence. Second, the ranges of doses studied varied widely between trials, limiting our ability to evaluate any specific dose intensity or compare lower doses to “target doses” recommended by guidelines. Additionally, for trials evaluating more than two dosages, we selected the highest and lowest daily doses for the intervention and control group, respectively, for our analyses. For instance, in one trial the higher-dose arm received enalapril 10 mg daily, which is a quarter of the maximum approved dose for this indication (40 mg) and half the recommended target dose, whereas in another the lower-dose arm received enalapril 20 mg daily (the recommended target dose) and the higher-dose arm received 60 mg daily (triple the target dose). Finally, we could not reliably assess for publication bias as there were too few studies (<10 trials) in each meta-analysis.[24]

## Conclusion

Evidence comparing lower and higher doses of drugs in HFrEF is sparse and imprecise. Higher doses of ACEIs and ARBs reduce the risk of HF worsening compared to lower doses, and higher doses of ARBs also reduce the risk of HF hospitalization. Effects of higher versus lower doses of these agents on other outcomes, as well as the impact of higher versus lower doses of BBs on any efficacy outcome, remain unclear. Higher doses importantly increase the risk of hypotension and agent-specific adverse effects (e.g. hyperkalemia, cough, creatinine rise) compared to lower doses. These results reinforce recommendations to start these agents at low doses, involve patients in dose titration decisions, carefully monitor for adverse effects after dose changes, and not be averse to lowering doses when tolerability issues arise.

## Supporting information

S1 Appendix (DOCX)
