# Supplementary material for: Higher versus lower doses of ACE inhibitors, angiotensin-2 receptor blockers and beta-blockers in heart failure with reduced ejection fraction: Systematic review and meta-analysis
Source: PLoS One. 2019 Feb 28;14(2):e0212907. doi: 10.1371/journal.pone.0212907 (PMC6394936; doi:10.1371/journal.pone.0212907)
Supplement: S1 Appendix — (DOCX) [file pone.0212907.s001.docx]

**Appendix 1: Search strategy for Ovid MEDLINE**

Ovid Medline In-Process & Other Non-Indexed Citations, Ovid MEDLINE Daily and Ovid MEDLINE 1946 to Present; search run April 25^th^, 2018.

| **#** | **Searches** |
| --- | --- |
| 1 | exp Heart Failure/ |
| 2 | (heart failure or chf or hfref or hfpef).tw. |
| 3 | 1 or 2 |
| 4 | exp Adrenergic beta-Antagonists/ |
| 5 | (beta blocker* or Acebutolol or Alprenolol or amosulalol or arotinolol or Atenolol or befunolol or Betaxolol or bevantolol or Bisoprolol or bopindolol or bromoacetylalprenololmenthane or bucindolol or bufetolol or bufuralol or Bunolol or Bupranolol or butofilolol or Butoxamine or carazolol or Carteolol or carvedilol or Celiprolol or cyanopindolol or Dihydroalprenolol or epanolol or esmolol or exaprolol or flestolol or indenolol or Iodocyanopindolol or Labetalol or landiolol or Levobunolol or medroxalol or mepindolol or Metipranolol or Metoprolol or Nadolol or nipradilol or Oxprenolol or Penbutolol or Pindolol or Practolol or prizidilol or Propranolol or Sotalol or talinolol or tertatolol or tilisolol or Timolol or tobanum).tw. |
| 6 | exp Angiotensin-Converting Enzyme Inhibitors/ |
| 7 | (acei or ace inhibitor* or angiotensin-converting enzyme inhibitor* or alacepril or benazepril or benazeprilat or Captopril or ceronapril or Cilazapril or cilazaprilat or delapril or Enalapril or Enalaprilat or Fosinopril or fosinoprilat or glyceraldehyde or imidapril or libenzapril or Lisinopril or LVV-hemorphin 6 or moexipril or omapatrilat or Perindopril or perindoprilat or quinapril or quinaprilat or Ramipril or ramiprilat or rentiapril or spirapril or temocapril hydrochloride or Teprotide or trandolapril or zofenopril).tw. |
| 8 | exp Angiotensin Receptor Antagonists/ |
| 9 | (arb or angiotensin receptor blocker* or Amlodipine or candesartan or enoltasosartan or eprosartan or irbesartan or Losartan or olmesartan or saprisartan or Saralasin or tasosartan or telmisartan).tw. |
| 10 | or/4-9 |
| 11 | Dose-Response Relationship, Drug/ |
| 12 | ((compar* or versus or vs) adj5 (dos* or high-dos* or low-dos*)).tw. |
| 13 | (dose dependen* or dose respons* or dose-ranging or dose ranging or dose finding or dose-finding or dos* effect* or target dos*).tw. |
| 14 | or/11-13 |
| 15 | (Randomized Controlled Trial or Pragmatic Clinical Trial).pt. or exp Randomized Controlled Trials as Topic/ or Randomized Controlled Trial/ |
| 16 | random allocation/ or double-blind method/ or single-blind method/ |
| 17 | (random* or sham or placebo*).mp. |
| 18 | ((singl* or doubl*) adj (blind* or dumm* or mask*)).mp. |
| 19 | ((tripl* or trebl*) adj (blind* or dumm* or mask*)).mp. |
| 20 | or/15-19 |
| 21 | 3 and 10 and 14 and 20 |
| 22 | limit 21 to english language |

*lines 15-20 adapted from *Strings attached: CADTH database search filters* [Internet]. Ottawa: CADTH; 2016. September 22, 2016. Available from: [www.cadth.ca/resources/finding-evidence](https://www.cadth.ca/resources/finding-evidence)
